# Relationship between Health Literacy, Health-Related Behaviors and Health Status: A Survey of Elderly Chinese

**DOI:** 10.3390/ijerph120809714

**Published:** 2015-08-18

**Authors:** Yong-Bing Liu, Liu Liu, Yan-Fei Li, Yan-Li Chen

**Affiliations:** 1 Nursing Department of Medical College, Yangzhou University, Yangzhou 225002, China; 2 Clinical Nursing Department of Nursing College, Xinjiang Medical University; Urumqi 830000, China; E-Mails: 15739078976@163.com (L.L.); lyf900105@126.com (Y.-F.L.); gl08290528@126.com (Y.-L.C.)

**Keywords:** health literacy, health-related behaviors, health status, nursing homes, relationship

## Abstract

*Background*: Despite the large volume of research dedicated to health-related behavior change, chronic disease costs continue to rise, thus creating a major public health burden. Health literacy, the ability to seek, understand, and utilize health information, has been identified as an important factor in the course of chronic conditions. Little research has been conducted on the relationship between health literacy and health-related behaviors and health status in elderly Chinese. The aim of this study was to elucidate the relationship between health literacy and health-related behaviors and health status in China. *Methods*: The subjects enrolled in this study were selected based on a stratified cluster random sampling design. Information involving >4500 older adults in 44 pension institutions in Urumqi, Changji, Karamay, and Shihezi of Xinjiang between September 2011 and June 2012 was collected. The Chinese Citizen Health Literacy Questionnaire (China Health Education Centre, 2008) and a Scale of the General Status were administered and the information was obtained through face-to-face inquiries by investigators. A total of 1452 respondents met the inclusion criteria. A total of 1452 questionnaires were issued and the valid response rate was 96.14% (1396 of 1452). Factors affecting health literacy and the relationship to health literacy were identified by one-way ANOVA and a multiple linear regression model. *Results*: The average health literacy level of the elderly in nursing homes was relatively low (71.74 ± 28.35 points). There were significant differences in the health literacy score among the factors of age, gender, race, education level, household income, marital conditions, and former occupation (*p* < 0.001). The health literacy score was significantly associated with smoking, drinking, physical exercise, and health examination (*p* < 0.001). The elderly with higher health literacy scores were significantly less likely to have risky behaviors (smoking, regular drinking, and lack of physical exercise), and in turn significantly more likely to undergo health examinations regularly, report good self-rated health, and significantly more likely to access sufficient health information from multiple sources (*p* < 0.001). No differences were noted between the health literacy score and BMI (*p* > 0.05). Multiple linear regression analysis showed that the independent influencing factors of health literacy included education level, race, former occupation, household income, age, physical exercise, health examination, smoking, and health information access (*p* < 0.001). *Conclusions*: Health literacy was significantly associated with health-related behaviors in elderly Chinese. Further longitudinal studies are needed to help confirm that improving health literacy in the elderly may be effective in changing health-related behaviors. To reduce risky habits, educational interventions to improve health literacy should be simultaneously conducted in health promotion work.

## 1. Introduction

The aging of China’s population is on the rise, and by the middle of this century will reach its peak. The elderly population >65 years of age is approximately 137.55 million [1], which accounts for approximately 10.1% of the total Chinese population. It is estimated that the proportion of elderly will increase to 16% in 2030 and to 23% in 2050 [2]. Thus, there will be one older adult among nearly every four Chinese on average, and there will be one older adult among every two-to-three people in big cities [3].

More than ever, older adults want to take control of their health [4,5] and rely less on health care providers as their only source of health information [6]. Health literacy has begun to attract notice in many countries, including China, which is known for its rapidly aging population.

Health literacy has been defined as “the degree to which individuals have the capacity to obtain, process, and understand basic health information and services needed to make appropriate health decisions” [7]. Health literacy has increased in importance in terms of public health and health promotion, as well as clinical care. Health literacy is now recognized as a key factor with respect to clinical “risk” and personal “assets” [8,9].

In December 2009, China’s Health Ministry published the results of the first official health literacy survey involving Chinese residents. Only 3.81% of older adults who belong to the 65–69 year old age group have adequate health literacy, which was the lowest level of all age groups surveyed [10]. Studies have revealed that health literacy is associated with health behavior [11] in the general population. To date, little research has been conducted regarding the relationship between health literacy and health-related behaviors and health status in elderly Chinese.

Because of the significant surge in growth of the elderly and the continued increase in the prevalence of chronic disease, there is a compelling need for studies to address the limited information on the health literacy and health-related behaviors of the elderly which currently exists. Thus, as healthy aging gains momentum as an essential tenet of current aging policy in China, it is critically important for policy makers to understand how best to encourage, support, and maintain positive health-related behaviors and health status among our growing number of older adults.

In this paper we present some of the first evidence on health literacy and health risk behaviors involving the Chinese elderly. We focus on health risks, including physical exercise, health examinations, alcohol consumption, and smoking [12], which are known to be responsible for a substantial proportion of morbidity and mortality in Chinese, as well as populations worldwide [13]. The aim of this study was to determine the relationship between health literacy and health-related behaviors and health status in Chinese adults 60–99 years of age.

## 2. Methods

### 2.1. Sample and Setting

The subjects enrolled in this study were selected based on the following criteria: (1) Sampling principles: A stratified cluster random sampling design was used for sampling. Information about >4500 elderly adults in 44 pension institutions in Urumqi, Changji, Karamay, and Shihezi of Xinjiang between September 2011 and June 2012 was collected. The information was obtained through face-to-face inquiry by investigators asking questions. (2) Inclusion criteria: (a) Age ≥ 60 years; (b) Clear consciousness, ability to read or communicate, and accessibility to investigators; and (c) The respondents agreed to cooperate with the investigators after the purpose of the research was explained. (3) Exclusion criteria: Mental disorders, cognitive disorders, severe, and end-stage diseases. A total of 1452 respondents met the aforementioned inclusion criteria. A total of 1452 questionnaires were issued and the valid response rate was 96.14% (1396 of 1452). Prior written and informed consent were obtained from every patient and the study was approved by the Ethics Review Board of Xinjiang Medical University.

### 2.2. Instruments

The two instruments used in this study were the Chinese Citizen Health Literacy Questionnaire (China Health Education Centre, 2008) [14] and a Scale of the General Status.

#### 2.2.1. Chinese Citizen Health Literacy Questionnaire

In this study the Chinese Citizen Health Literacy Questionnaire was used [14]. The questionnaire includes four dimensions (belief literacy, knowledge literacy, behavior literacy, and skill literacy). These four dimensions contain a total of 98 items, and the score for each item is 2 points, making the maximum number of points 196. This questionnaire has a Cronbach α coefficient of 0.904, indicating high reliability and good content validity; thus, the Chinese Citizen Health Literacy Questionnaire satisfactorily reflects the health literacy level of senior citizens.

#### 2.2.2. Scale of the General Status

The survey instrument adapted standardized and validated questions across several domains, including health-related behaviors, health status, and sociodemographic characteristics (including name, age, gender, race, education, household income, family size, marital status, and occupation).

### 2.3. Measures

#### 2.3.1. Health Literacy

Among the 98 entries in the questionnaire, the lowest score was 0 points and the highest score was 2 points. For questions about belief literacy, wrong answers were scored 0 points, neutrality was scored 1 point, and correct answers were scored 2 points. “Do not know” answers were regarded as wrong answers and were scored 0 points. For questions about knowledge literacy, behavior literacy, and skill literacy, wrong answers were scored 0 points and correct answers were scored 2 points. The “do not know” answers were regarded as wrong answers and were scored 0 points. According to the scoring criteria, the scores of belief literacy, knowledge literacy, behavior literacy, and skill literacy were calculated. The total score of health literacy was calculated by adding the scores of the four dimensions with equal weighting. A higher score indicated a higher level of health literacy [14].

#### 2.3.2. Health-Related Behaviors

Health-related behaviors include health behavior and health information access.

##### Health Behavior

Health behavior was assessed in terms of smoking, drinking, physical exercise, health examinations, and body mass index (BMI) [12]. These items, together with dietary habits, are counted among important health behaviors to be improved in the national health promotion movement and secular changes in these items in the Chinese population have been monitored through regular national surveys. Respondents chose one of 3–4 options in response to each question. For analysis, the original 3–4 categories were dichotomized in accordance with the most commonly provided advice on smoking, drinking, physical exercise, and health examinations in public health settings in China, as follows: “quit smoking;” “have at least two alcohol-free days every week;” “get regular physical exercise of moderate-to-vigorous intensity;” and “undergo annual health examinations.” The smoking categories were “smoker” (currently smoke) *versus* “non-smoker” (have quit smoking/have never smoked). The drinking categories were “daily” (drink almost every day) *versus* “not daily” (drink on a weekly basis/on a monthly basis/rarely). The physical exercise categories were “weekly” (do more than twice a week/on a weekly basis) *versus* “not weekly” (do on a monthly basis/rarely). The health examination categories were “annually” (undergo every year) *versus* “not annually” (undergo once per 2 years/once per 3–4 years/once per 5 years or less) [15].

The BMI was calculated as the weight in kilograms divided by the height in meters squared. Based on BMI values, respondents were categorized into the following four groups: (1) underweight (BMI ≤ 18.5 kg/m^2^); (2) normal (18.5 kg/m^2^ < BMI ≤ 24.0 kg/m^2^); (3) overweight (24.0 kg/m^2^ < BMI ≤ 28.0 kg/m^2^); and (4) obese (BMI > 28.0 kg/m^2^).

##### Health Information Access

Participants were asked whether or not they could acquire the health information they wanted, and answered on a 4-point scale (certainly yes/probably yes/probably no/certainly no). The participants who gave affirmative answers “certainly yes” and “probably yes” were considered as having “sufficient” access to health information. This item probably indicated an overall satisfaction rating for the outcome of information-seeking. In the absence of established measures, we defined this item as a key indicator of health information access [15]. Participants were also asked how frequently they used the following sources to seek health information: (1) hospitals; (2) pharmacies; (3) healthcare facilities; (4) family and friends; (5) books and magazines; (6) radio and television; and (7) the Internet. Respondents chose one of four options (often/sometimes/seldom/very seldom). The number of sources that were used “often” or “sometimes”: was counted for each respondent. In the information age, health information-seekers choose the most appropriate sources for the subject matter according to their preference and ability [16,17,18,19]. The participants who have access to more sources have a greater likelihood of accessing relevant information. We considered the number of sources as an important component of information-seeking behavior which could affect the outcome of information-seeking.

#### 2.3.3. Health Status

Participants were asked to rate their current health status on a 5-point scale (excellent/good/moderate/poor/very poor). For analysis, responses were trichotomized into “good” (excellent/good), “moderate” (moderate), and “poor” (poor/very poor) [15].

### 2.4. Data Analysis

A database was established using EXCEL2000. Double-entry and validation was conducted. SPSS15.0 was used for data analysis. Measurement data were presented as *x* ± *s*. Enumeration data were expressed as a rate or constituent ratio. A t-test was used for comparing differences between two groups. One-way ANOVA was used for comparing the differences among multiple groups. An equation of elderly health literacy was established using a multiple linear regression model (*a*_in_ = 0.05, *a*_out_ = 0.10). A *p* < 0.05 was considered to be statistically significant.

## 3. Results

### 3.1. The Relationship between Elderly Health Literacy Levels and Sociodemographic Characteristics

To determine the health literacy status of older adults, the association between sociodemographic characteristics and elderly health literacy levels were analyzed. The sociodemographic characteristics and the health literacy scores of the older adults are shown in Table 1. The older adults in this study were 60–99 years of age, with an average age of 77.37 ± 8.48 years. The percentage of older adults 75–84 years of age was 51.86%. There were 619 males and 777 females, accounting for 44.34% and 55.66% of the study group, respectively. The majority of older adults were Han population (94.2%). The education level was mostly primary school or below, and older adults with this education level accounted for 61.53% of the study group. The majority of older adults had a household income of 2000–5000 yuan/month, which accounted for 45.85% of the study group. The percentage of older adults with a family size of 3–5 persons was 51.36%, which was higher than other family sizes. With respect to marital status, the widowed population accounted for 70.56% of the study group. Older adults who listed farming as a former occupation accounted for 34.96% of the study group.

The mean health literacy score was 71.74 ± 28.35 (range, 0–196). One-way ANOVA was used to compare the differences among the factors of age, gender, race, education level, household income, family size, marital status, and former occupation. There were significant differences in health literacy scores as a function of age, gender, race, education level, household income, marital status, and former occupation (*p* < 0.05; Table 1).

**Table 1:** Correlation between health literacy levels and sociodemographic characteristics in 1396 of older adults.

Sociodemographic Characteristics	Percentage (%)	Health literacy score ($\bar{x}\pm s$)	*F/t* Value	*p* Value
**Age (years)**			6.457	0.000
<65	102 (7.31)	74.13 ± 27.10		
65~74	311 (22.28)	76.21 ± 28.71		
75~84	724 (51.86)	71.53 ± 27.97		
≥85	259 (18.55)	65.99 ± 28.58		
**Gender**			3.522	0.000
Male	619 (44.34)	74.72 ± 28.80		
Female	777 (55.66)	69.36 ± 27.78		
**Race**			4.125	0.000
Han	1315 (94.20)	72.51 ± 27.81		
Minority	81 (5.80)	59.20 ± 33.77		
**Education level**			67.907	0.000
Primary school or below	859 (61.53)	64.05 ± 24.67		
Junior high school	220 (15.76)	79.24 ± 28.91		
Senior high school and technical secondary school	193 (13.83)	84.98 ± 26.95		
Graduate and above	124 (8.88)	91.04 ±32.93		
**Household income (Yuan/month)**			9.982	0.000
< 500	62 (4.44)	57.52 ± 27.53		
500~1000	76 (5.44)	66.11 ± 26.44		
1000~2000	474 (33.95)	68.54 ± 28.73		
2000~5000	640 (45.85)	74.74 ± 27.22		
≥ 5000	144 (10.32)	78.04 ± 29.85		
**Family size (Person)**			0.647	0.524
1~2	266 (19.05)	73.49 ± 31.47		
3~5	717 (51.36)	71.45 ± 27.49		
≥6	413 (29.58)	71.10 ± 27.73		
**Marriage status**			6.904	0.000
Unmarried	38 (2.72)	71.50 ± 28.88		
Married	327 (23.42)	76.73 ± 29.22		
Widowed	985 (70.56)	69.65 ± 27.82		
Divorced	46 (3.30)	81.07 ± 27.64		
**Former Occupation**			32.609	0.000
Manager	218 (15.62)	86.30 ± 31.71		
Ordinary staff	121 (8.67)	67.97 ± 29.63		
Professionals	237 (16.98)	80.86 ± 26.03		
Service industry employee	101 (7.23)	66.97 ± 30.62		
Production staff	231 (16.55)	73.44 ± 28.08		
Farmers	488 (34.96)	61.92 ± 22.43		

### 3.2. The Relationships of the Health Literacy Score with Health-Related Behaviors and Health Status

The relationships of the health literacy score with health-related behaviors and health status are shown in Table 2. The health literacy score was significantly associated with smoking, drinking, physical exercise, and health examinations (*p* < 0.001). Those who had sufficient available health information had higher health literacy scores than those who had insufficient available health information. The participants who used a greater number of sources to seek health information had higher health literacy scores and were more likely to have sufficient available health information (*p* < 0.001). Moreover, the relationship between the health literacy score and health status was significant; specifically, the participants who had higher health literacy scores were significantly more likely to report good self-rated health (*p* < 0.001). Interestingly, no differences were noted between health literacy score and BMI (*p* > 0.05).

**Table 2:** Relationships of the health literacy score with health-related behaviors and health status.

Variables	N	Mean ± SD	*F/t* Value	*p*
**Available health information**			9.988	0.001
Insufficient	1066 (76.36)	67.66 ± 26.73		
Sufficient	330 (23.64)	84.90 ± 29.45		
**Number of sources**			31.92	0.001
0	195 (13.97)	60.58 ± 25.70		
1	451 (32.31)	65.67 ± 28.12		
2	501 (35.89)	73.49 ± 24.85		
3	192 (13.75)	86.33 ± 28.83		
≥4	57 (4.08)	98.68 ± 30.95		
**Smoking**			−4.622	0.001
Nonsmoker	1072 (76.79)	78.07 ± 29.21		
Smoker	324 (23.21)	69.82 ± 27.82		
**Drinking**			−4.328	0.001
Not daily	1175 (84.17)	79.26 ± 32.35		
Daily	221 (15.83)	70.32 ± 27.32		
**Physical exercise**			−7.154	0.001
Not weekly	500 (35.82)	64.60 ± 26.19		
Weekly	896 (64.18)	75.72 ± 28.74		
**Health examination**			6.428	0.001
Not Annually	361 (25.86)	63.60 ± 26.78		
Annually	1035 (74.14)	74.58 ± 28.35		
**BMI categories**			0.727	0.536
<18.5	146 (10.46)	69.60 ± 28.65		
18.5~24.0	572 (40.97)	73.06 ± 26.48		
24.0~28.0	474 (33.95)	75.64 ± 29.51		
≥28.0	204 (14.62)	73.75 ± 27.38		
**Health status**			5.726	0.003
Good	545 (39.04)	74.86 ± 29.08		
Moderate	580 (41.55)	70.20 ± 28.24		
Poor	271 (19.41)	68.73 ± 26.59		

### 3.3. Multivariable Analysis of Correlates of Health Literacy with Sociodemographic Characteristics, Health-Related Behaviors, and Health Status

To determine the relationship of health literacy with sociodemographic characteristics, health-related behaviors, and health status, multiple linear regression analysis was used. With respect to sociodemographic characteristics, the independent influencing factors for health literacy were education level, race, former occupation, household income, and age (Table 3).

Individuals with a high school education or more compared with those without a high school degree were significantly more likely to have high health literacy. Older adults who were white collar workers or had professional occupations tended to have higher health literacy scores than blue collar workers or laborers. Older adults with a high income level were likely to have high health literacy.

With respect to health-related behaviors, the independent influencing factors for health literacy were physical exercise, health examinations, smoking, and health information access. Older adults who engaged in more physical exercise, had health examinations annually, were non-smokers, and had sufficient access to health information had higher health literacy scores.

**Table 3:** Multiple linear regression analysis about health literacy with sociodemographic characteristics, health behavior, health status.

Variables		Partial Regression Coefficient	SE	Standardized Partial Regression Coefficient	*t*	*p*
X4	Education level	6.717	0.786	0.239	8.550	0.001
X 12	Physical exercise	9.288	1.411	0.158	6.583	0.001
X 13	Health examination	6.999	1.512	0.109	4.628	0.001
X 8–1	Manager	12.970	2.216	0.168	5.852	0.001
X 10	Smoking	−6.544	2.074	−0.098	−3.155	0.002
X 3	Race	−16.472	3.024	−0.134	−5.448	0.001
X 9	health information	7.824	1.690	0.118	4.628	0.001
X 8–3	Professionals	8.583	2.096	0.114	4.095	0.001
X 8–5	Production stuff	6.416	2.029	0.085	3.162	0.002
X 1	Age	−0.256	0.084	−0.077	−3.070	0.002
X 5	Household income	2.351	0.761	0.076	3.088	0.002

## 4. Discussion

The mean health literacy score was 71.74 ± 28.35 for older adults, which is a low level. Studies to date involving the health literacy of Chinese senior citizens have mostly focused on subjects 60–69 years of age [14]. Few studies have involved older subjects and no studies have reported the health literacy of the elderly who are institutionalized. Compared with the results of Zhao *et al.* [20] (average age, 54.27 ± 6.80 years; total health literacy score, 108.37 ± 28.85), senior citizens in Xinjiang had a lower level of health literacy (average age, 79.18 ± 8.81 years; total health literacy score, 71.74 ± 28.35). Thus, more attention should be paid to the health literacy education of this population.

Li *et al.* [14] showed that health literacy was correlated to region, gender, age, educational level, marital status, family size, average monthly income, and occupation. Paasche-Orlow *et al.* [21] reported that a low health literacy level was correlated to education level, race, and age. The findings of this study are basically in agreement with previous studies. We must pay more attention to the elderly population.

This study examined the relationship between health literacy and health-related behaviors and health status in Chinese 60–99 years of age. There have been few study that have focused on the impact of health literacy in relation to health promotion [22]. The research involving the relationship between health literacy and health-related behaviors has just begun in the context of public health and health promotion in China. Further longitudinal studies are needed to demonstrate that health literacy intervention is an important aspect of health promotion. Despite some limitations, the findings of these efforts suggest that health literacy may play a key role in health promotion, especially in the large elderly population countries, such as China.

The participants with higher health literacy scores were significantly less likely to have risky behavior (smoking, regular drinking, and lack of physical exercise), and in turn significantly more likely to report health examinations regularly, good self-rated health, and sufficient access to health information from multiple sources. An interview investigation of the British population 18–90 years of age showed that high functional health literacy was associated with being a non-smoker, adequate fruit and vegetable consumption, and good self-rated health independent of age, gender, ethnicity, education, and income [22]. Although the instruments for measuring health literacy vary across studies, the findings of this survey are basically consistent with previous population-based studies [22,23,24].

This study provides the first step toward understanding the relationships connecting health literacy with health-related behaviors in elderly Chinese. A systematic review revealed that interventions to improve health literacy in primary health care and community settings may be effective in changing health behavior [25]. To reduce risky habits, educational interventions to improve health literacy should be simultaneously conducted in health promotion efforts.

The current study had a number of potential limitations. Multiple linear regression analysis showed that health status was not the independent influencing factor of health literacy. This study depended on self-report measures, which is prone to measurement errors and missing values. While self-rated health can reflect objective health status and indicate a comprehensive index of health status, self-rated health is not always equal to objective health status at the individual level. Moreover, the overall level of health literacy scores of the Chinese elderly population were very low, and the direct impact of health literacy on health status was not statistically significant. This result was inconsistent with previous studies [26] that have demonstrated that health literacy is associated with health status. Further studies are needed to elucidate in more detail the mechanisms linking health literacy to health status.

Indeed, health literacy plays an important role in health promotion. Therefore, health professionals should pay more attention to the impact of health literacy on health behaviors. To promote the effects of health promotion interventions, we should improve the health literacy levels of older adults.

## 5. Conclusions

In this study we showed a significant association between health literacy and health-related behaviors in elderly Chinese. Improving health literacy in the older population may be effective in changing health-related behaviors. The mean health literacy score was low, thus health professionals should pay more attention to the status quo. To reduce risky habits, educational interventions to improve health literacy should be simultaneously conducted in health promotion efforts. Teamwork should be applied to improve health literacy of older adults. A teach-back method should be used in health literacy intervention in which health professionals provide professional services and training skills to assist in meeting the inadequate health literacy of older adults.
